# Determination of Renal Distribution of Zinc, Copper, Iron, and Platinum in Mouse Kidney Using LA-ICP-MS

**DOI:** 10.1155/2021/6800294

**Published:** 2021-10-26

**Authors:** Petr Stepka, Monika Kratochvilova, Michaela Kuchynka, Martina Raudenska, Hana Holcova Polanska, Tomas Vicar, Tomas Vaculovic, Marketa Vaculovicova, Michal Masarik

**Affiliations:** ^1^Department of Physiology, Faculty of Medicine, Masaryk University, Kamenice 5, CZ-625 00 Brno, Czech Republic; ^2^Department of Chemistry, Faculty of Science, Masaryk University, Kotlarska 2, CZ-611 37 Brno, Czech Republic; ^3^Masaryk University, Faculty of Pharmacy, Department of Chemical Drugs, Palackeho 1-3, CZ-612 42 Brno, Czech Republic; ^4^Department of Pathological Physiology, Faculty of Medicine, Masaryk University, Kamenice 5, CZ-625 00 Brno, Czech Republic; ^5^Department of Biomedical Engineering, Faculty of Electrical Engineering and Communication, Brno University of Technology, Technicka 3058/10, CZ-616 00 Brno, Czech Republic; ^6^Institute of Laboratory Research on Geomaterials, Faculty of Natural Sciences, Comenius University in Bratislava, Mlynska dolina, Ilkovicova 6, SK-842 15 Bratislava, Slovakia; ^7^Central European Institute of Technology, Brno University of Technology, Technicka 3058/10, CZ-616 00 Brno, Czech Republic; ^8^Department of Chemistry and Biochemistry, Mendel University in Brno, Zemedelska 1, CZ-61300 Brno, Czech Republic

## Abstract

The main dose-limiting side effect of cisplatin is nephrotoxicity. The utilization of cisplatin is an issue of balancing tumour toxicity versus platinum-induced nephrotoxicity. In this study, we focused on intraorgan distribution of common essential trace elements zinc, copper, and iron in healthy mouse kidneys and distribution of platinum after cisplatin treatment. Renal distribution in 12 nontreated Nu-Nu mice (males) was assessed by laser ablation inductively coupled plasma mass spectrometry (LA-ICP-MS). Furthermore, 9 Nu-Nu mice were treated with cisplatin. The order of elements concentration in kidneys was as follows: Fe > Zn > Cu. All three metals showed the higher concentrations at the cortex and medulla (28.60, 3.35, and 93.83 *μ*g/g for Zn, Cu, and Fe, respectively) and lower concentration at the pelvis and the urinary tract (20.20, 1.93, and 62.48 *μ*g/g for Zn, Cu, and Fe, respectively). No statistically significant difference between cortex and medulla was observed for these elements. After platinum treatment, the concentration of platinum in kidneys was enhanced more than 60-times, *p* < 0.001. Platinum significantly showed the highest accumulation in cortex (2.11 *μ*g/g) with a gradient distribution. Platinum was less accumulated in medulla and pelvis than in cortex, and the lowest accumulation occurred in the urinary tract (1.13 *μ*g/g). Image processing has been successfully utilized to colocalize metal distribution using LA-ICP-MS and histological samples images.

## 1. Introduction

Some metals are essential for cell homeostasis. Zinc, iron, and copper are common essential elements and participate in the regulation of numerous physiological processes such as protein synthesis, enzymatic reactions, and antioxidant defences. These metals are useful at very low concentrations but can be toxic in larger amounts or certain forms [1]. The kidneys are prone to be damaged by metal toxicity, because of their capability to absorb and accumulate divalent metals [2, 3]. Chronic and acute metal exposure has been shown to induce nephropathies with varied grades of seriousness ranging from tubular dysfunctions to life-threatening renal failure [4, 5].

Compounds containing metals have been utilized in medicine for decades, and platinum-containing chemotherapeutic agents remain key components for the treatment of various types of cancer [6]. The main dose-limiting side effect of such platinum derivatives is nephrotoxicity [2, 7]. Due to the kidney function complexity (excretory function, management of electrolyte and acid–base balance, regulation of blood pressure, etc.), the utilization of cisplatin is an issue of balancing tumour toxicity versus platinum-induced nephrotoxicity [8].

For the studies of the effect of metals on the kidney, it is important to know how the levels of metals vary within different histological regions [9]. In the kidney, we can distinguish an outer cortex region and an inner medullar region. The cortex and medulla drain into the hollow pelvis, the funnel-shaped beginning of the ureter. Consequently, the kidney is characterized by a histological complexity, containing a plenty of specialized cell types.

The method of laser ablation inductively coupled plasma mass spectrometry (LA-ICP-MS) is a useful tool for determination of element distribution in various samples. It has been utilized for quantitative imaging of platinum group elements in tumor spheroids [10], determination of cisplatin retention in cochlea after chemotherapy [11], or for quantification of the interactions of platinum compound with human serum albumin [12].

In this study, we focused on the intraorgan distribution of zinc, copper, and iron in healthy mouse kidneys and distribution of platinum after cisplatin treatment by using LA-ICP-MS combined with routine microscopic techniques. Metal distribution maps obtained using LA-ICP-MS and histological samples images of the organ were correlated via the utilization of image processing.

## 2. Materials and Methods

### 2.1. Animal Experiments

The use of the animals followed the European Commission Guidelines. The experiments were performed with the approval of the Ethics Commission at the Faculty of Medicine, Masaryk University, Brno, Czech Republic. Renal distribution of zinc, copper, and iron was assessed in 12 eight-week-old nontreated Nu-Nu male mice (weight 25–30 g). Furthermore, 9 Nu-Nu male mice (3 control and 6 with induced tumours) were treated with cisplatin. Tumours were induced by subcutaneous injection of suspension with 10^5^ PC-3 cells. The PC-3 prostate cancer cell line was derived from bone metastasis of a 4-grade prostatic adenocarcinoma of a 62-year-old Caucasian male and was purchased from HPA Culture Collections (Salisbury, UK). Mice received four doses of cisplatin (Cisplatin 1 mg/mL concentrate for solution for infusion, Ebewe, Austria) intraperitoneally within two weeks with the concentration of 0.5 mg/mL in volume 40 *μ*L/10 g of mouse weight. Each application was followed by a two-day pause. This concept tries to simulate treatment regimen of cancer patients. The resulting concentration and distribution of platinum in kidneys were assessed by LA-ICP-MS.

### 2.2. Sample Preparation

The animals were terminated two days after the last dose of cisplatin. Next, kidneys were extracted and embedded in CryoGlue Embedding medium (SLEE Medical, Mainz, Germany) and put inside liquid nitrogen for 10 s. Consequently, samples were cut on a Cryostat MTC (SLEE Medical) with thickness of 25 *μ*m and 5 *μ*m for LA-ICP-MS analysis and hematoxylin-eosin staining, respectively. Slices have been electrostatically adhered to SuperFrost Plus glass slides (Thermo Scientific, Germany) and fixed by air-drying. Neighbouring slices were always used for microscopy analysis. Part of the organ (typically 0.1 g of kidney) was used in analyses for accessing elements distribution in the homogenized sample. The concentration of metal was related to the weight of a particular tissue sample of a particular animal.

### 2.3. Determination of Element Distribution in Kidney

The determination of the distribution of elements of interests in cuts of the kidney was performed using LA-ICP-MS. All ablation experiments were done using a commercial laser ablation system UP 213 (New Wave, USA) consisting of Q-Switch Nd:YAG laser source emitting radiation with a wavelength of 213 nm and a pulse width of 4.2 nm and a moveable ablation cell (SuperCell™, washout time 1.04 s). Ablated material in the ablation cell was transported by helium with a flow rate of 1.0 L min^−1^ and introduced into the ICP. A quadrupole ICP-MS 7500ce (Agilent Technologies, Japan) instrument equipped with a collision cell, operated in He-mode (2.5 mL min^−1^) for minimization of possible interferences by polyatomic ions, was employed. Argon (0.6 L min^−1^) was admixed with the sample aerosol before entering the ICP torch.

The following isotopes were measured with given integration times ^12^C (0.01 s), ^28^Si (0.1 s), ^56^Fe (0.1 s), ^65^Cu (0.1 s), ^66^Zn (0.1 s), and ^195^Pt (0.1 s). Laser ablation parameters influencing resolution (laser beam diameter and scan speed) were optimized according to reference [13] to get sufficient lateral resolution in a sufficiently short-time analysis. The laser spot diameter was 100 *μ*m, and the scan speed was 200 *μ*m/s. Laser beam parameters as fluence and laser repetition rate were 8 J/cm^2^ and 10 Hz, respectively. The high laser beam fluence was applied to compensate different ablation rate in various zones of the sample [14]. Samples and standards were ablated in line mode. The standard was ablated by one line, whereas the sample was ablated by the series of individual lines.

### 2.4. Preparation of Standards for Calibration

0.1 g of agarose powder (Agarose MP, Roche) was mixed with 5 mL miliQ water and known addition of standard reference solution Astasol (Analytika, Ltd.). This solution was heated to form agarose gel and pipetted on the quartz slide and let to dry (at room temperature) to form a thin film. Agarose gels were treated with known amounts of elements of interest (Fe, Cu, Zn, and Pt) to obtain a set of standards with 0, 10, 50, and 100 mg kg^−1^ of Fe, Cu, and Zn and 0, 2, 5, and 10 mg kg^−1^ of Pt.

The standards were ablated under the same laser ablation conditions as the samples (laser spot diameters of 100 *μ*m, scan speed of 200 *μ*m, laser beam fluence of 8 J/cm^2^, and repetition rate of 10 Hz).

The different ablation rate was compensated by the ablation of the whole thickness of the sample; therefore, no normalization was necessary during imaging. As published in reference [14], a signal of ^28^Si was used to monitor reaching the glass substrate with a consequent increase of Si signal. This increase indicates that whole thickness of sample was ablated.

### 2.5. Determination of Total Element Concentration

The determination of the total concentration of elements of interests in decomposed kidney samples was performed by solution nebulization ICP-MS (a 7500ce ICP-MS spectrometer from Agilent Technologies, Japan, was employed). For this purpose, 0.1 g of kidney sample was weighted; 5 mL of HNO_3_ (Analpure, Analytika, Ltd.) was added. Microwave-assisted digestion (ETHOS 1; Milestone, Italy) was used for decomposition. The procedure consisted of two steps: (i) gradual increase in temperature from the room temperature up to 200°C over 15 min and (ii) 20 minutes at 200°C with the maximum input power at 1000 W. Before the analysis, the samples were diluted by factor 10. The solution was nebulized by Babington nebulizer coupled to a Scott double-pass spray chamber. The ICP-MS operating parameters were optimized with respect to getting the highest signal/noise ratio, minimal formation of oxide, and double-charged ions; the carrier gas (argon) flow rate was set to 0.75 L min^−1^, and the makeup gas (argon) flow rate was 0.40 L min^−1^. The sample uptake rate was 0.33 mL·min^−1^. The signals of monitored isotopes (^56^Fe, ^65^Cu, ^66^Zn, and ^195^Pt) were measured with an integration time of 0.1 s in five replicates. The quantification was done in two steps. In the first one, external calibration was used for semiquantitative determination of Fe, Cu, Zn, and Pt. This semiquantitative step was performed to allow for subsequent compensation of the matrix effect. Consequently, quantitative determination by standard addition was performed to correct the sample matrix effect. The results obtained for standard addition calibration were in accordance with results of semiquantitative analysis.

### 2.6. Image Processing and Statistics

In order to determine the metal distribution in different regions of histological samples, manually drawn regions of interest have been identified in histological images. For computation of metals in each region, it is necessary to register (i.e., align) images of the distribution of metals to the histological image. Real scale of both distribution of metals and the histological image is known, but simple rescaling to the same scale provides unsatisfactory results, thanks to different translation and rotation during sample placement. Additionally, compensation for compression of the sample during slicing is needed. For compensation of these deformations, we have used image registration techniques in order to align the images.

Because of multimodal nature of the images, mutual information [15] was used as registration metric. Mutual information is defined as

(1) $$\begin{array}{c} I_{\text{AB}}=H_{\text{A}}+H_{\text{B}}-H_{\text{A},\text{B}},\\ H_{\text{A}}=-\underset{i}{\sum }p\left(a_{i}\right)\log p\left(a_{i}\right),\\ H_{\text{A},\text{B}}=-\underset{j}{\sum }\underset{k}{\sum }p\left(a_{j,}b_{k}\right)\log\;p\left(a_{j,}b_{k}\right), \end{array}$$

where *H*_A_, *H*_B_ is information entropy of images A and B, respectively, *H*_A,B_ is joint entropy of images A and B. Marginal and joint probabilities (approximated by histograms) are labelled as *p*(*a*), *p*(*b*), and *p*(*a*, *b*), respectively. We used an affine geometric transformation *T* defined as

(2) $$\begin{array}{c} u=\left[\begin{array}{c} u\\ v\\ 1 \end{array}\right]=T\;x=\left[\begin{array}{c} \begin{array}{ccc} a & b & c\\ d & e & f \end{array}\\ 0\;0\;1 \end{array}\right]\left[\begin{array}{c} x\\ y\\ 1 \end{array}\right], \end{array}$$

where [*x*, *y*]  and [*u*, *v*] are old and new pixel coordinates, respectively. Parameters of *T* must be estimated, where it allows translation, rotation, scaling, and sheering. For estimation of geometric transformation matrix to register to histological image, image of distribution of Fe was used, because it is evenly distributed in the whole sample. The transformation estimated for Fe image was used to transform other metal images. This method produced some errors when metals were inappropriately distributed. These errors were corrected manually by adding several control points to unsatisfactory images. For manual correction, the same affine transformation was used, but optimized criteria were defined as a sum of Euclidian distances between corresponding control points, which were manually selected in both images, i.e.,

(3) $$\begin{array}{c} E\left(T\right)=\underset{i}{\sum }{\left\|u_{i}^{*}-{Tx}_{i}^{*}\right\|}^{2}, \end{array}$$

where *u*_*i*_^∗^ = [*u*_*i*_^∗^, *v*_*i*_^∗^, 1]^*T*^ is a position of *i*th control point in metal distribution image, *x*_*i*_^∗^ = [*x*_*i*_^∗^, *y*_*i*,_^∗^1]^*T*^ is a corresponding position control point in histological image, and ‖∙‖ is the Euclidean (L2) norm. As is shown in reference [16], we can construct a system of linear equations from all points and solve for *T* using Moore-Penrose pseudoinverse:

(4) $$\begin{array}{c} U=T\;X,\\ T=U\left({\left(X^{T}X\right)}^{-1}X^{T}\right), \end{array}$$

where for construction of matrices *U* = [*u*_1_^∗^, *u*_2_^∗^, ⋯, *u*_*n*_^∗^] and *X* = [*x*_1_^∗^, *x*_2_^∗^, ⋯, *x*_*n*_^∗^] at least 3 control points are needed to make the problem solvable.

Finally, a manually drawn mask of regions in histological images was used to determine concentration values of the investigated metals to build their distribution images. Due to nonnormal distributions of these metals in the mask regions, which is caused by inaccurate labelling, the median of metal concentration values was rather used for the statistical analysis. Data were analysed using paired tests ANOVA in Matlab 2017a software (MathWorks, Natick, MA, USA).

## 3. Results and Discussion

### 3.1. Distribution of Zinc, Copper, and Iron in Mouse Kidneys

The Fe, Zn, and Cu concentrations in homogenized kidney tissue samples was assessed by solution nebulization ICP-MS. The concentration order of these elements in the homogenized kidney samples was as follows: Fe > Zn > Cu.

**Figure 1:**
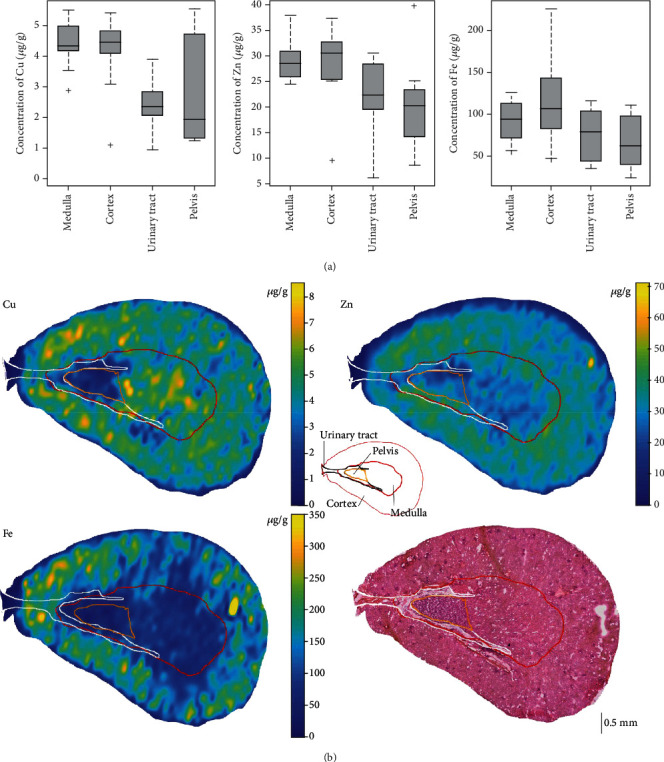
Distribution of Fe, Zn, and Cu in kidney. (a) Distribution of metals in mineralised (decomposed) regions of kidney, (b) metal distribution maps obtained using LA-ICP-MS, and light microscopy image of a representative sample.

In order to obtain the spatial distribution of these metals, LA-ICP-MS was performed and combined with light microscopy images of hematoxylin-eosin samples. Image processing has been utilized to colocalize LA-ICP-MS maps with histological sample image. Cortex, medulla, pelvis, and the urinary tract were assessed separately. According to Figures 1(a) and 1(b), all three metals were more concentrated in the cortex and medulla (average value of 28.60, 3.35, and 93.83 *μ*g/g for Zn, Cu, and Fe, respectively) compared to the pelvis and the urinary tract (20.20, 1.93, and 62.48 *μ*g/g for Zn, Cu, and Fe, respectively). No statistically significant difference between cortex and medulla as well as between pelvis and urinary tract was observed for these elements.

### 3.2. Platinum Distribution in Mouse Kidney after Cisplatin Treatment

**Figure 2:**
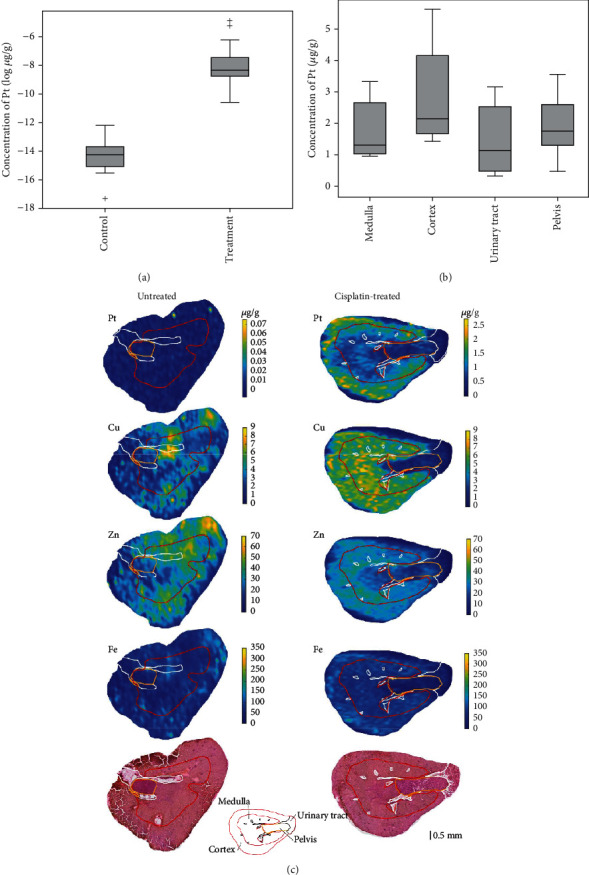
Platinum distribution in kidneys of mouse untreated or treated with cisplatin. (a) Concentration of Pt in mineralized (decomposed) kidney samples before and after treatment with cisplatin. (b) Distribution of Pt in histological and mineralised regions of kidney, (c) metal distribution maps obtained using LA-IC/-MS and light microscopy image of a representative sample.

After treatment with cisplatin, the concentration of platinum in the mouse kidneys was enhanced more than 60-times (*p* < 0.001), as depicted by Figure 2(a). According to Figures 2(b) and 2(c), Pt accumulation was higher in the cortex and showed a concentration gradient. The highest Pt concentration was found in the cortex (2.11 *μ*g/g, *p* < 0.001) and the lowest one in the urinary tract (1.13 *μ*g/g, *p* = 0.004). Further representative tissue sections can be seen in Supplementary Figure 1.

### 3.3. Comparison of Healthy and Platin-Treated Mouse Kidneys

The comparison between individual samples of kidney was done based on the ratio of elements of interest concentrations. The main focus was on Pt as a chemotherapeutic agent and Cu, because copper transporter Ctr1 is responsible for Pt uptake [17], together with Zn, which has been implicated in affecting cisplatin metabolism via the upregulation of metallothioneins [18]. In this experiment, kidneys from mouse of the control group (without tumour induced by PC-3 cancer cells and without cis-Pt application) were analysed, and Cu and Zn concentrations ranged from 0.1 to 1.0 mg/kg and 0.7 to 12 mg/kg, respectively. The influence of application of PC-3 and cisplatin was accessed as the ratio of Pt/Cu, Pt/Zn, and Zn/Cu.

When the Zn/Cu ratio values are compared, in Table 1, it is seen the ratio varies from 4.0 (treated with cisplatin) to 7.0 (nontreated with cisplatin) for mouse that did not receive PC-3 and from 1 (nontreated with cisplatin) to 7.0 (treated with cisplatin) for mouse that received PC-3.

The Pt/Cu, Pt/Zn, and Zn/Cu concentration ratios were also measured by scanning the sample using LA-ICP, and an increased ratio of Pt/Cu and Pt/Zn was found in the outermost part of kidneys. In case of Pt/Cu the ratio raised up to 4, whereas the Pt/Zn ratio is significantly lower (up to 0.5).

## 4. Discussion

Nephrotoxicity is one of the major side effects of chemotherapy with metal-based drugs. It is associated with considerable morbidity and mortality. The renal accumulation of metals is greater than in other organs, because kidneys are a major route for their excretion [2, 3]. Regardless of considerable efforts to discover less-toxic alternatives, cisplatin is still widely prescribed in clinical practice. However, the cisplatin-induced side effects are severe. LA-ICP-MS has been previously used for example to determine cisplatin retention in cochlea after chemotherapy, which can lead to hearing-loss side effects [11]. The utilization of cisplatin can often be an issue of balancing tumour toxicity versus platinum-induced nephrotoxicity [8]. In this sense, quantitative imaging of metal distribution can elucidate the storage and behaviour of metals in that organ. In this study, LA-ICP-MS proved to be suitable for this type of analyses. The order of measured elements concentrations in kidneys was as follows: Fe > Zn > Cu, which is in accordance with previous findings in human kidney [19]. Copper transporter Ctr1 is highly expressed in proximal and distal tubular cells in mouse kidneys [17] and has been also demonstrated to mediate the cellular uptake of cisplatin. Since copper concentration was shown to influence cisplatin uptake [17, 20], the similar representation of iron, zinc, and copper in human and mouse kidneys support the usability of mouse model for analyses of platinum distribution in the kidney. Zinc, copper, and iron concentrations were higher in the cortex and medulla and lower in the pelvis and the urinary tract. Livingston found the zinc concentration decreased from the outer surface of the cortex to the inner medullary surface [21], which is in accordance with results obtained in the present study, even though the difference between the investigated element concentrations in cortex and medulla was not statistically significant.

With the use of image processing, it was possible to colocalize element distribution maps obtained by LA-ICP-MS with corresponding histological sample images for precise localization of elements in regions of the kidney.

After platinum treatment, the concentration of platinum in kidneys was enhanced more than 60 times. Platinum accumulation was as follows: cortex > medulla > pelvis > urinary tract. The prevailing cortical localization of platinum after cisplatin treatment is in good accordance with previous studies [2, 22–25]. On the other hand, Zhang et al. assumed that renal medulla is more sensitive to cisplatin than cortex [9]. Cisplatin is freely filtered at the glomeruli and taken up by renal tubular cells [26]. Both cortex and medulla could be impaired by cisplatin. Nevertheless, damaged cells were mainly detected in renal cortex and outer medulla and not in inner medulla [27]. In this region, the organic cation transporter-2 (Oct-2), an important mediator of the cellular uptake of cisplatin, is highly expressed, with a significantly higher expression in male rodents [25, 28–31].

In conclusion, based on the results of the present study and the facts about cisplatin treatment, it can be assumed that structures present in cortex such as proximal convoluted tubule, glomerulus, and distal convoluted tubule could be severely damaged by cisplatin during cancer treatment. Furthermore, the heterogeneous distribution of Fe, Zn, Cu, and Pt in the kidney indicated that cautious sampling and precise localization of measured elements within the organ is needed in any comparative study about elements distribution in kidney. More precise localization can be achieved by combining the use of LA-ICP-MS with light microscopy and image processing.

## Figures and Tables

**Table 1:** Summarization of Pt/Cu, Pt/Zn, and Zn/Cu median concentration ratios in homogenized and mineralized kidney samples and concentration range found using LA-ICP-MS (in parenthesis).

Treatment	Pt/Cu	Pt/Zn	Zn/Cu
Nonreceiving PC-3 and nontreated with cisplatin	—	—	7 (5-20)
Nonreceiving PC-3 and treated with cisplatin	0.6 (0.25-2)	0.2 (0.08-0.4)	4 (2-18)
Receiving PC-3 and nontreated with cisplatin	—	—	1 (4-20)
Receiving PC-3 and treated with cisplatin	0.9 (0.4-4)	0.2 (0.05-0.5)	7 (3-13)

## Data Availability

The data that support the findings of this study are available upon reasonable request.

## Supplementary Materials

Supplementary Materials Supplementary Figure 1: metal distribution maps obtained using LA-IC/-MS and light microscopy image of a representative sample.
