# Supplementary figures and images for: Determination of Renal Distribution of Zinc, Copper, Iron, and Platinum in Mouse Kidney Using LA-ICP-MS
Source: Biomed Res Int. 2021 Oct 26;2021:6800294. doi: 10.1155/2021/6800294 (PMC8564192; doi:10.1155/2021/6800294)

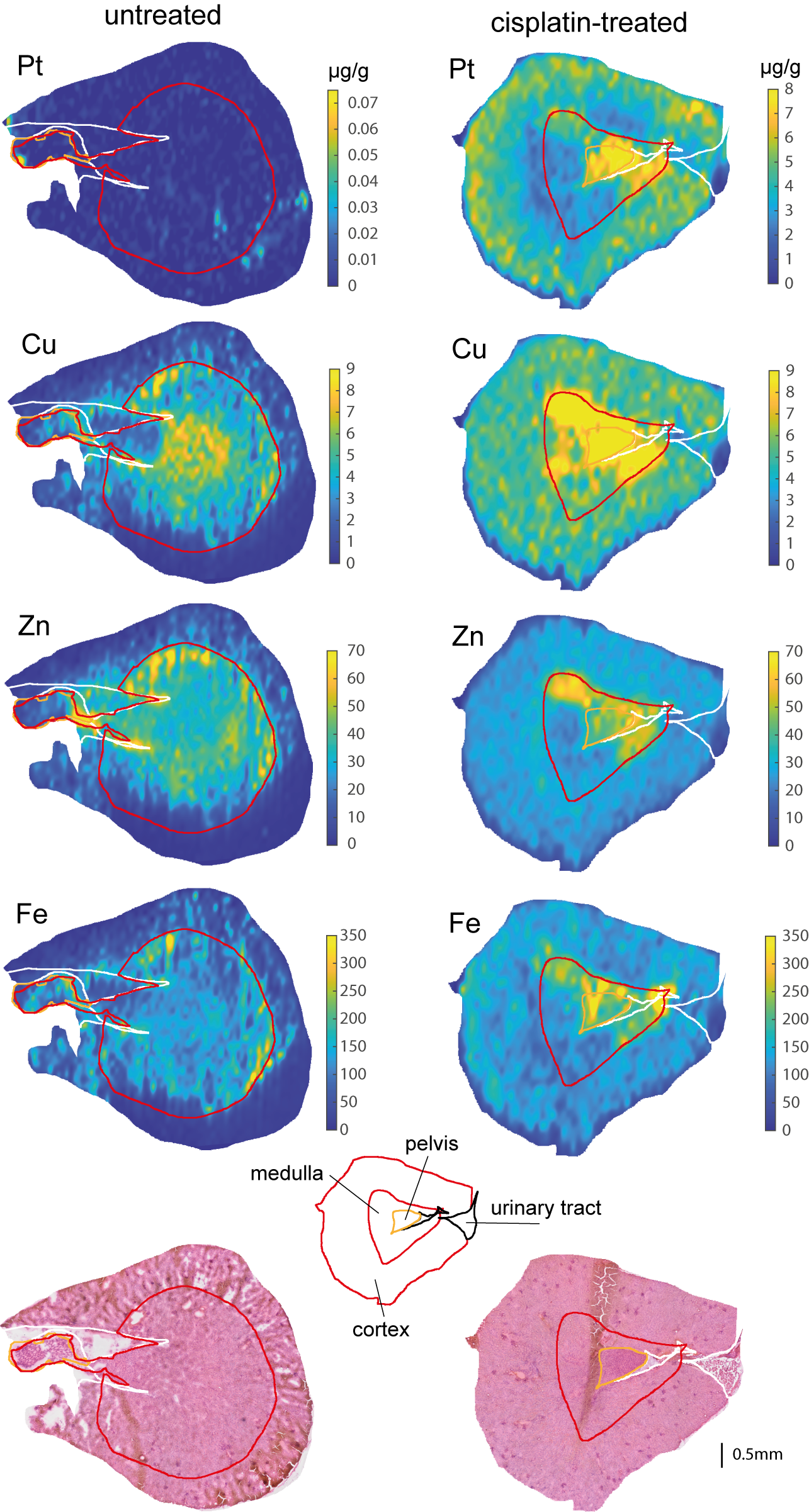

Supplement: Supplementary Materials — Supplementary Figure 1: metal distribution maps obtained using LA-IC/-MS and light microscopy image of a representative sample. [file 6800294.f1.jpeg]
